# Calcitriol confers neuroprotective effects in traumatic brain injury by activating Nrf2 signaling through an autophagy-mediated mechanism

**DOI:** 10.1186/s10020-021-00377-1

**Published:** 2021-09-23

**Authors:** Changmeng Cui, Changshui Wang, Feng Jin, Mengqi Yang, Lingsheng Kong, Wenxiu Han, Pei Jiang

**Affiliations:** 1 Department of Neurosurgery, Affiliated Hospital of Jining Medical University, Jining Medical University, Jining, China; 2 Institute of Clinical Pharmacy & Pharmacology, Jining First People’s Hospital, Jining Medical University, Jining, 272011 China

**Keywords:** Traumatic brain injury, Vitamin D, Autophagy, Keap-Nrf2 system

## Abstract

**Background:**

The present study aimed to further explore the potential interaction between oxidative stress and autophagy in the progression of traumatic brain injury (TBI) and therapeutic mechanism of calcitriol, the active form of vitamin D (VitD).

**Methods:**

Neuroprotective effects of calcitriol were examined following TBI. We further evaluated the impacts of TBI and calcitriol treatment on autophagic process and nuclear factor E2-related factor 2 (Nrf2) signaling.

**Results:**

We found that treatment of calcitriol markedly ameliorated the neurological deficits and histopathological changes following TBI. The brain damage impaired autophagic flux and impeded Nrf2 signaling, the major regulator in antioxidant response, consequently leading to uncontrolled and excessive oxidative stress. Meanwhile, calcitriol promoted autophagic process and activated Nrf2 signaling as evidenced by the reduced Keap1 expression and enhanced Nrf2 translocation, thereby mitigating TBI-induced oxidative damage. In support, we further found that chloroquine (CQ) treatment abrogated calcitriol-induced autophagy and compromised Nrf2 activation with increased Keap1 accumulation and reduced expression of Nrf2-targeted genes. Additionally, both CQ treatment and Nrf2 genetic knockout abolished the protective effects of calcitriol against both TBI-induced neurological deficits and neuronal apoptosis.

**Conclusions:**

Therefore, our work demonstrated a neuroprotective role of calcitriol in TBI by triggering Nrf2 activation, which might be mediated by autophagy.

**Supplementary Information:**

The online version contains supplementary material available at 10.1186/s10020-021-00377-1.

## Background

Traumatic brain injury (TBI) is one of the most common causes of death and long-term impairment that affects all ages (Stout et al. 2018). The brain damage is induced by both primary and secondary injury processes. The primary injury is the mechanical disruption of brain tissue that occurs immediately, whereas secondary injury subsequently develops over time via intricate processes, including excitotoxicity, neuroinflammation and oxidative stress that ultimately contribute to neuronal loss (Ma et al. 2018). Additionally, TBI may cause rupture of cerebral blood vessels, induce blood clots and increase blood–brain barrier permeability. These dysfunctions would further exacerbate the hypoxia condition, resulting in more serious oxidative damage (Vázquez-Rosa et al. 2020). Because of its complexity, effective therapies for the treatment of TBI are still inadequate.

Autophagy is a major proteolytic system that senses intracellular stressful conditions and rapidly mounts a molecular response to deal with the damage through sequestration and degradation of dysfunctional organelles and compromised proteins (Zhang et al. 2019a). The autophagic process is orchestrated through a series of autophagy-related genes (ATG genes), such as LC3-II (derived from LC3-I upon lipidation) and Beclin-1, which are verified biomarkers for evaluating autophagy. In addition, the adaptor protein p62 serves as a cargo receptor responsible for recognizing and loading ubiquitinated proteins into autophagosomes for degradation in selective autophagy (Su et al. 2019). Notably, p62 also activates nuclear factor E2-related factor 2 (Nrf2) signaling, a well-characterized cellular defense mechanism against oxidative stress, by interacting with Kelch-like ECH-associated protein 1 (Keap1) (Saito et al. 2020). In the cytoplasm, Nrf2 is bound to its partner, Keap1, and is rapidly degraded through the ubiquitin–proteasome pathway (Zheng et al. 2020a). It has been demonstrated that p62 directly interacts with Keap1, thereby dissociating it from Nrf2 and directing it toward autophagic degradation (Deng et al. 2020; Dodson et al. 2015).

Vitamin D (VitD) is recognized as a novel neuroactive steroid, which has antioxidant and neuroprotective activities in addition to its classical function in bone metabolism and calcium-phosphate homeostasis (Cui et al. 2019). VitD is either obtained from the diet or photosynthesized from 7-dehydrocholesterol in the skin upon ultraviolet light exposure. After two steps of hydroxylation by P450 enzymes (25-hydroxylase and 1α-hydroxylase, respectively), the active form of VitD, 1,25-dihydroxyvitamin D_3_ (calcitriol), is generated (Jiang et al. 2014). Through binding with VitD receptor (VDR), calcitriol exerts multiple aspects of pathophysiological actions (Cui et al. 2019). We previously demonstrated that calcitriol treatment is effective in preventing TBI-induced behavioral abnormalities and oxidative stress (Cui et al. 2017a). Moreover, calcitriol treatment also promotes autophagic flux, preventing autophagosome accumulation following TBI (Cui et al. 2017b). Emerging evidence has indicated that the antioxidant activity of VitD is tightly related to Keap1/Nrf2 signaling (Zheng et al. 2020b; Nachliely et al. 2019), suggesting that VDR activation may mediate the interaction between autophagy and the Keap1/Nrf2 system to facilitate the neuroprotective functions of calcitriol.

The present study aims to test the protective actions of calcitriol following TBI and its impacts on autophagy and Nrf2 signaling. Moreover, through blocking autophagic process by using chloroquine (CQ) and genetic knockout of Nrf2, we further sought to confirm the hypothesis that autophagy may interact with Keap1/Nrf2 system to contribute to the neuroprotective effects of calcitriol.

## Methods

### Animal care and drug treatment

Adult male CD1 Elite mice (22–28 g, 10–12 weeks; Jining, China) were used in this study. Nrf2 knockout (*Nrf2*^*−/−*^) CD1 male mice were obtained from Dr. Xin Guo (Hebei Medical University). The Institutional Animal Care and Use Committee of Jining Medical University approved all the experiments, which were performed according to the guidelines of the National Institutes of Health Guide for the Care and Use of Laboratory Animals (NIH Publications No.80-23). The mice were housed under standard conditions and were randomly assigned among one of five groups: sham, TBI, TBI + low dose (0.5 µg/kg) of calcitriol, TBI + medium dose (1 µg/kg) of calcitriol, and TBI + high dose (3 µg/kg) of calcitriol. The animals received daily gavage of vehicle or different doses of calcitriol for 14 days. Moreover, to confirm the neuroprotective mechanisms of calcitriol, CQ (30 mg/kg/day) was co-treated with calcitriol by intraperitoneal injection to block autophagy flux.

### Mouse model of TBI

A 10-mm-long midline scalp incision was made to expose the skull. We then used a high-speed surgical hand drill to create a 2 × 2-mm^2^ craniotomy (0.5 mm rostral and lateral from bregma) over the right frontal cortex with the dura intact. With a 2.5-mm-diameter rounded metal tip, the controlled cortical impact (CCI) device (CCI Model 6.3; Custom Design, USA) induced a moderate-severe TBI onto the exposed brain at a velocity of 4 m/s, with a depth of 2 mm and a contact time of 200 ms. The bone flap was immediately replaced and sealed, and the scalp was sutured. Since the present study mainly focused on the TBI recovery process and the neuroprotective effects of calcitriol, the frontal cortex around the injured area were used for biochemical analysis after the behavioral test.

### Neurological score evaluation

At 1–14 days following TBI, the neurological scores were determined as Neurological Severity Scores, a composite of motor, sensory, reflex, and balance tests (normal score: 2–3; maximal deficit score: 18; Additional file 1).

### The Morris water maze (MWM) test

The apparatus consisted of a circular black water tank (180 cm in diameter, 50 cm high) filled with water (26 °C) to a depth of 30 cm. An escape platform (diameter 12 cm, height 28 cm, painted opaque) submerged 2 cm below the water surface was placed in the middle of one of the quadrants equidistant from the tank wall and the center of the pool. All the mice were trained to find the platform before the sham operation or the induction of TBI. The orientation navigation test was performed by evaluating the escape latency at 11–14 days after TBI. For each trial, each mouse was randomly placed into a quadrant start point (N, S, E, or W) facing the wall of the pool and was allowed a maximum of 60 s to find the escape platform. The mice that failed to escape within 60 s were placed on the platform for a maximum of 20 s and returned to their cage to await a new trial (intertrial interval, 10 min). On the final day, the mice were subjected to a spatial probe test 4 h after the last orientation navigation trials, in which the platform was removed. The time spent in the target quadrant and the swimming speed were evaluated.

### Histopathological staining

After fixation, the brains were embedded in paraffin and sliced into 4-µm coronal sections at the level of the bregma and stained with hematoxylin and eosin (H&E). Apoptosis was assessed using terminal deoxynucleotidyl transferase-mediated cyanine–dUTP nick-end labeling (TUNEL) following the manufacturer’s protocol. Nuclei were counterstained with DAPI (Beyotime Biotechnology, China). For each group, histopathological changes in frontal cortex from three different mice were used for quantification.

### Transmission electron microscopy (TEM)

To observe the autolysosomes in neuronal cells, tissues from frontal cortex were immersed in 2% glutaraldehyde and 1% osmium tetroxide (Sigma-Aldrich; Merck KGaA) for 2 h at 4 °C, and then dehydrated via a graded ethanol series. Following the displacement of ethanol with propylene oxide, the tissues were embedded in Epon (both from Sigma-Aldrich) and sectioned along the coronal plane with a diamond knife (FernAnclez-hIorln 1953; Ivan Sorvall, Inc., New York, NY, USA) at a thickness of 60 nm. The sections were stained with lead citrate and observed using a CM-120 electron microscope (Philips, Eindhoven, Netherlands). Neuronal cells were characterized by a large, euchromatic (pale-staining) nucleus, abundant rough endoplasmic reticulum, mitochondria, and Golgi complexes in the cytoplasm, while glial cells were characterized with a smaller cell body and heterochromatic nucleus stained more intensely than the nucleus of the neuron. In order to quantify the alteration of the number of the autolysosomes, the area of the cell cytoplasm was measured by using Image-Pro Plus 6.0.

### Western blot analysis

Proteins (50 µg) were separated by 12% SDS–PAGE and transferred onto a nitrocellulose membrane. The membrane was blocked with 5% nonfat milk at room temperature for 2 h and then incubated overnight at 4 °C with primary antibodies against microtubule-associated protein light chain 3 (LC3) (1:500, PM036, MBL, Nagoya, Japan), p62 (1:1,000, ab101266, Abcam, Cambridge, MA, USA), beclin 1 (1:1,000, ab137161, Abcam), Keap1 (1:1,000, ab118285, Abcam), Nrf2 (1:1,000, ab62352, Abcam), beta-actin (1:1,500, A1978, Sigma, USA), and PCNA (1:500, ab92552, Abcam). The gray values of the protein bands were measured using ImageJ software and normalized to that of β-actin that was used as an internal control.

### Real-time PCR analysis

Total RNA was extracted using Trizol reagent (Invitrogen, USA) following the manufacturer’s instructions. Quantitative PCR was performed on a Bio-Rad Cx96 Detection System (Bio-Rad, USA) using a SYBR green PCR kit (Applied Biosystems, USA) and gene-specific primers (Additional file 2).

### Immunofluorescence staining

Frozen cross-sections (15 µm) were prepared and examined. The sections were incubated with primary antibodies against Keap1 (1:200, ab139729), p62 (1:200, ab56416), and Nrf2 (1:1,000, ab62352) (all from Abcam) overnight at 4 °C, and then with a mixture of FITC- and TRITC-conjugated secondary antibodies for 2 h at room temperature. Images were captured under a fluorescence microscope (Olympus, Japan). Finally, the fluorescence intensity was quantified using ImageJ software. The negative control is a secondary-only assay to demonstrate low non-specific binding of the secondary antibody (Additional file 3).

### Detection of oxidative parameters

The generation of reactive oxygen species (ROS) was determined by fluorescence-labeled dihydroethidium (DHE). Frozen cross-sections (15 µm) were incubated in DHE for 30 min at 37 °C in a dark humidified chamber. The sections were rinsed three times in PBS and observed using an inverted fluorescence microscope (Olympus, Japan). Malondialdehyde (MDA) levels were measured using the thiobarbituric acid reactive substances (TBARS) assay. The activities of superoxide dismutase (SOD), catalase (CAT), and glutathione (GSH) were determined using SOD, CAT, and GSH assay kits, respectively (Nanjing Jiancheng Bioengineering Institute, China).

### Primary cortical neuron culture

The skull, blood and meninges were carefully removed from fetal mouse brains. After the cortical tissue from frontal cortex was digested in 0.25% trypsin (BI, Israel) for 5 min at 37 °C, the suspensions, containing fetal bovine serum (BI, Israel), were passed through filters with a 0.22-μm pore size (Millipore, USA) and then centrifuged at 1500 rpm for 5 min. The cells were distributed in poly-d-lysine-coated plates. The medium was replaced with neurobasal medium supplemented with streptomycin, penicillin, HEPES, glutamate, and B27 (BI, Israel). The cells were exposed to different doses of calcitriol (1 nM, 10 nM, 100 nM, 500 nM) for 24 h. The cells were pre-treated for 6 h with 25 μM CQ and then treated with the indicated doses of calcitriol.

### Cell viability analysis

MTT was added to each well of a 24-well plate followed by incubation at 37 °C for 1 h. The purple formazan crystals formed through the reduction of MTT were then dissolved in 500 µL DMSO, and the absorbance of the wells was recorded at 590 nm. Cell viability was calculated by the absorbance ratio of the treated group to that of the control.

### Autophagic flux analysis

Autophagic flux was detected by using the RFP-GFP-LC3 adenovirus (Hanbio, China). After plating the cells in a 24-well plate at a density of 1 × 10^4^ cells/dish and incubating with mRFP-GFP-LC3 adenovirus for 24 h. Autophagic flux was observed under an inverted fluorescent microscope (Olympus, Japan). The yellow puncta indicated autophagosomes, and the red puncta indicated autolysosomes.

### Statistical analysis

The results were expressed as means ± SEM. All the analyses were performed using SPSS 17.0 software. Normality of distribution was assessed by the Lilliefors test and homogeneity of variance was tested with the Levene’s test. Statistical significance was determined using one-way analysis of variance (ANOVA), and the Student–Newman–Keuls post hoc test was used to determine differences among different groups. For variables that did not follow a Gaussian distribution or did not fulfill equal variance requirements, nonparametric Kruskal–Wallis analysis with the Dunn multiple comparisons was used. A *P*-value < 0.05 was considered statistically significant.

## Results

### The effects of calcitriol on TBI-induced neurological deficits

**Fig. 1:**
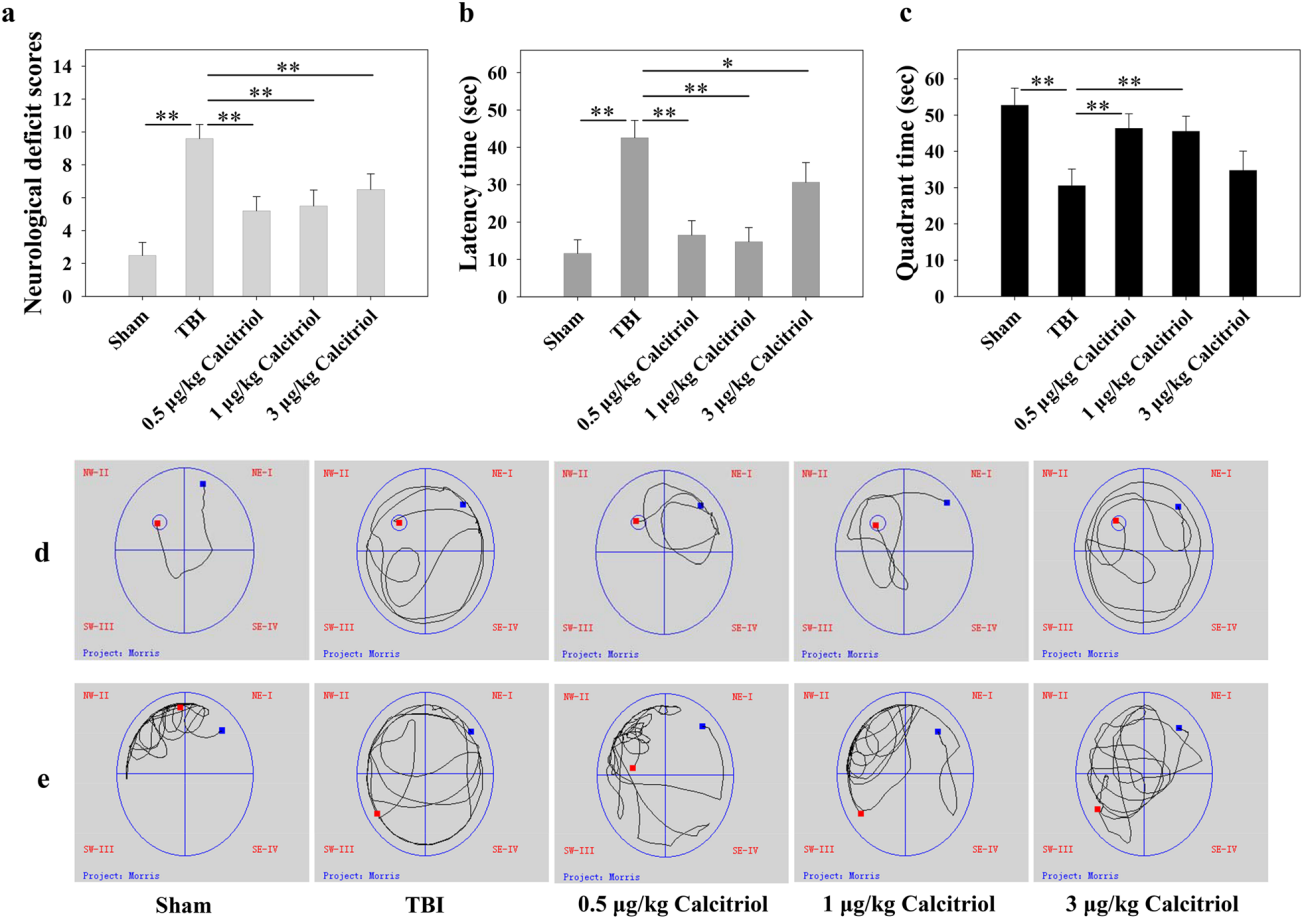
The neuroprotective effects of calcitriol in TBI-induced neurological deficits and memory dysfunction. **a** The variation in neurological deficits at 14 days after treatment was determined by neurological severity score tests. **b** The time (seconds) spent finding the submerged platform at 14 days. **c** The time (seconds) spent on exploring the quadrant that initially contained the platform at 14 days. **d** Representative traces indicating the sample paths of the mice in the orientation navigation test. **e** Representative traces in the probe test indicating the sample paths of the mice after the platform was removed. Data are presented as means ± SEM (*n* = 10). *P < 0.05 and **P < 0.01 versus the indicated groups. (Sham: sham-operated group; TBI: TBI model group; Calcitriol: TBI + calcitriol treatment group)

The neurological severity scores were assessed at 1–14 days after TBI (Additional file 4). At 14 days, the level of neurological injury was significantly increased in the TBI group compared with that in sham-operated animals (*P* < 0.01). In addition, compared with the TBI model group, TBI + calcitriol treatment group showed improved neurological deficit scores (*P* < 0.01). No significant differences were found among the mice treated with different calcitriol doses (Fig. 1a).

### The effects of calcitriol on TBI-induced learning and memory ability impairment

The MWM hidden platform task was used to investigate whether calcitriol could improve the spatial memory deficits at 11–14 days after TBI (Additional file 5a). As shown in Fig. 1b, compared with the sham-operated group, mice with TBI spent longer searching for the hidden platform at 14 days post-surgery (*P* < 0.01). In contrast, mice in TBI + calcitriol treatment group displayed a markedly shorter latency time compared with that in the TBI-only treatment group (*P* < 0.01 for the low-dose and medium-dose groups; *P* < 0.05 for the high-dose group). In the probe trials (Fig. 1c), mice with TBI spent less time than their sham-operated counterparts swimming toward the goal quadrant that previously contained the platform (*P* < 0.01). Nevertheless, mice in the low-dose and medium-dose calcitriol treatment groups displayed improved learned bias (*P* < 0.01), whereas the high-dose group failed to markedly improve the performance in the probe trial (Fig. 1d, e). No significant differences in swimming speeds were found among the groups, indicating that the observed differences were not a result of an inability to execute the swimming task (Additional file 5b).

### The effects of calcitriol on neuronal cell death after TBI

**Fig. 2:**
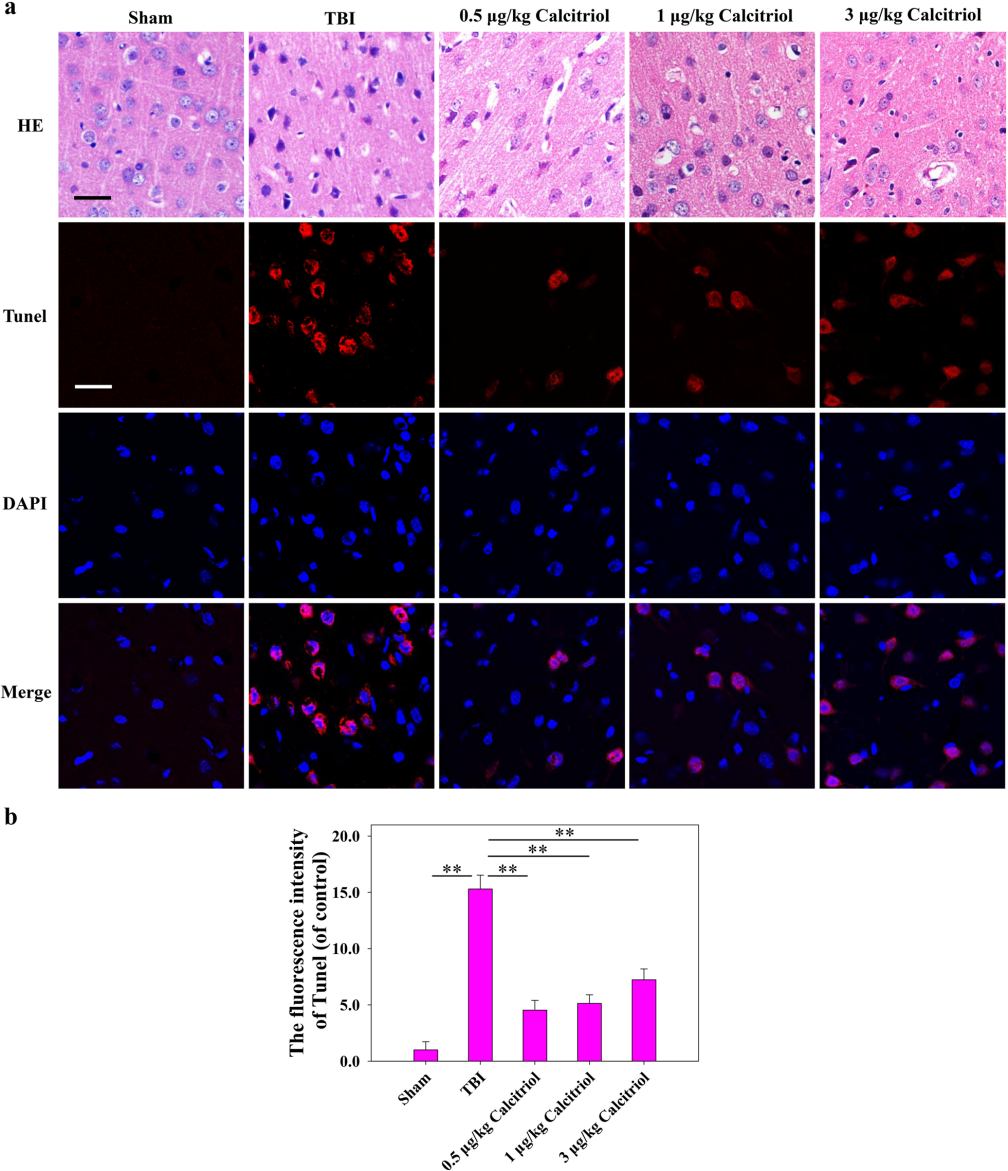
Calcitriol treatment mitigated TBI-induced histopathological changes. **a** Representative images of the histological assessment of the frontal cortex via hematoxylin and eosin (H&E) and TUNEL staining (Bar = 50 µm). **b** Statistical graphs of apoptosis cells (% of DAPI). Data are presented as means ± SEM (*n* = 5). **P < 0.01 versus the indicated groups. (Sham: sham-operated group; TBI: TBI model group; Calcitriol: TBI + calcitriol treatment group)

As depicted in Fig. 2, the frontal cortical neurons in the sham-operated group were arranged in an orderly manner, the cytoplasm was transparent, the cell nuclei showed a round or oval shape, the chromatin was evenly distributed, and the nucleoli were clear. After TBI, the opposite morphology was observed. In the calcitriol treatment group, the amount and level of neuronal degeneration, necrosis, and loss were significantly attenuated. Likewise, only a small number of TUNEL-positive neurons were found in the sham-operated group. However, the neuronal cells in the TBI model group were arranged in a disorderly manner, and a greater number of apoptotic neurons were observed (*P* < 0.01). After calcitriol treatment, the number of apoptotic cortical neurons was significantly decreased (*P* < 0.01). It should be noted that 0.5 µg/kg calcitriol is comparable or more effective than the higher doses. To decrease the possibility of the side effects of calcitriol such as hypercalcemia, the relatively low but effective dose, 0.5 µg/kg of calcitriol, was used in the following in vivo research to explore the molecular mechanism.

### The effects of calcitriol on TBI-induced autophagy impairment

**Fig. 3:**
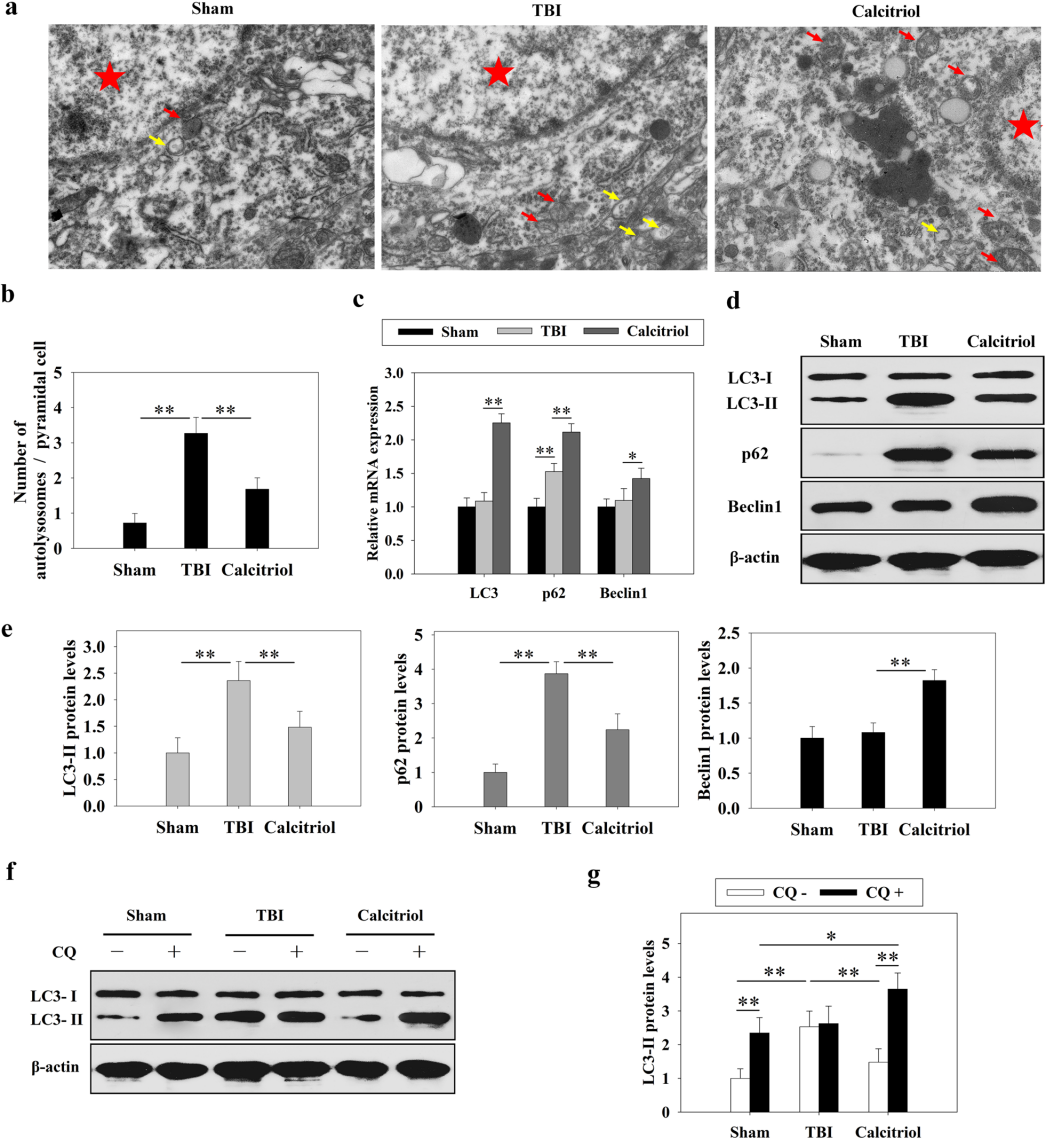
Calcitriol (0.5 µg/kg) treatment attenuated TBI-induced autophagic dysfunction. **a** Representative transmission electron microscopy (TEM) images of three groups. Red pentagram denotes representative nucleus, yellow arrows denote representative autophagosomes and red arrows denote representative autolysosomes. **b** Statistical graphs of number of autophagosomes and autolysosomes per cell. **c** The effects of calcitriol treatment and TBI on the mRNA expression levels of autophagic markers. **d** Representative images of Western blots of autophagic markers. **e** Graphs of LC3-II, p62, and beclin 1 protein expression levels. **f**, **g** Chloroquine (CQ) was used to evaluate the effects of calcitriol treatment and TBI on autophagic flux. Data are presented as means ± SEM (*n* = 5). *P < 0.05 and **P < 0.01 versus the indicated groups. (Sham: sham-operated group; TBI: TBI model group; Calcitriol: TBI + calcitriol treatment group)

TEM analysis demonstrated that in the TBI model group, there was a markedly increased accumulation of autophagosomes around the nucleus than in the sham-operated group (*P* < 0.01) (Fig. 3a). However, in TBI + calcitriol treatment group, the autophagosome abundance was reduced, whereas the autolysosomes were increased (*P* < 0.01 versus the TBI-only group). Moreover, we found that *LC3* and beclin 1 mRNA levels were unchanged after TBI, whereas that of p62 was markedly increased (*P* < 0.01). The mRNA expression levels of *LC3*, p62, and beclin 1 were all markedly higher in the calcitriol treatment group than in the TBI model group (*P* < 0.01 for LC3 and p62; *P* < 0.05 for beclin 1) (Fig. 3c). Intriguingly, TBI induced the protein expression of LC3-II and p62 (*P* < 0.01), but this effect was partly reversed following repeated calcitriol treatment (*P* < 0.01). However, no significant changes in beclin 1 protein expression were observed in the TBI model group, whereas beclin 1 was upregulated after calcitriol treatment (*P* < 0.01) (Fig. 3d, e). In addition, as shown in Fig. 3f and g, blocking autophagosome–lysosome fusion with CQ did not lead to an additional increase in LC3-II protein levels in TBI model group. These results suggested that TBI induced autophagy dysfunction and impaired autophagosome clearance, which was mitigated by calcitriol treatment.

### The effects of calcitriol on the Keap1-Nrf2 pathway

**Fig. 4:**
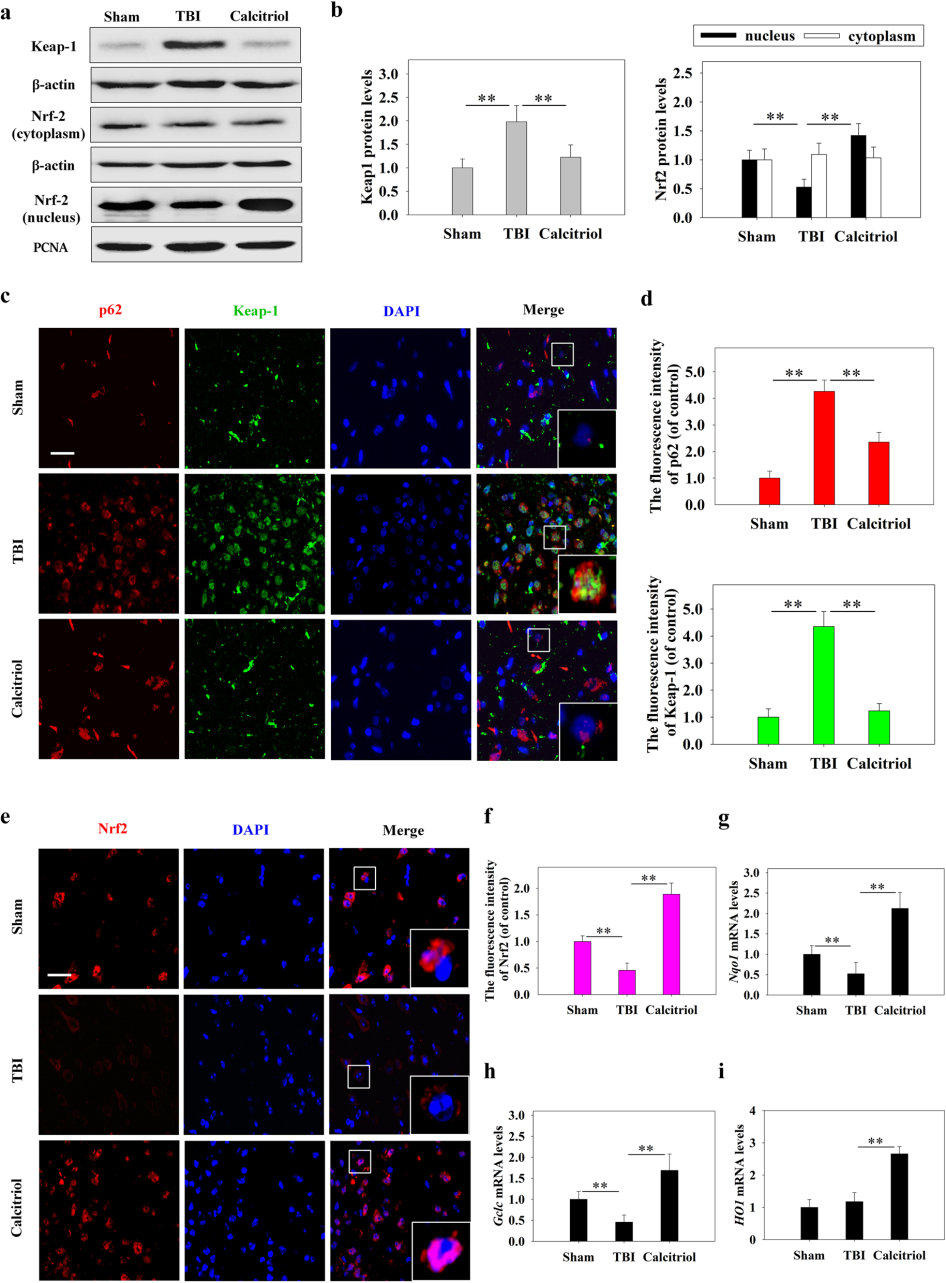
The effects of calcitriol (0.5 µg/kg) on Nrf2 signaling after TBI. **a** Representative images of Western blot staining for Keap1, cytoplasmic Nrf2, and nuclear Nrf2. **b** Statistical graphs of Keap1 protein expression and Nrf2 translocation. **c**, **d** Representative immunofluorescence images and statistical graphs of p62 and Keap1 co-expression (Bar = 50 µm). **e**, **f** Representative immunofluorescence images and statistical graphs of Nrf2 translocation (Bar = 50 µm). **g** The relative mRNA expression level of *Nqo1*. **h** The relative mRNA expression level of *Gclc*. **i** The relative mRNA expression level of *HO1*. Data are presented as means ± SEM (*n* = 5). *P < 0.05 and **P < 0.01 versus the indicated groups. (Sham: sham-operated group; TBI: TBI model group; Calcitriol: TBI + calcitriol treatment group)

As we found that calcitriol treatment could rescue TBI-induced autophagic flux dysfunction, we further investigated whether autophagic process affected the Keap1–Nrf2 pathway. The expression levels of Keap1 and Nrf2 were measured by Western blotting (Fig. 4a, b). The level of Keap1 was higher in the TBI model group than in the sham-operated group (*P* < 0.01); however, calcitriol treatment partially reduced the Keap1 expression level (*P* < 0.01). Immunofluorescent staining revealed similar changes in the protein levels of p62 and Keap1 (Fig. 4c, d). TBI led to a significant reduction in the expression of Nrf2 in the nucleus (*P* < 0.01), while calcitriol treatment increased the nuclear translocation of Nrf2 (*P* < 0.01). The mRNA levels of Nrf2 target genes (NADPH quinone dehydrogenase 1 [*Nqo1*], heme oxygenase 1 [*HO1*], glutamate-cysteine ligase catalytic subunit [*Gclc*]) were then evaluated by qPCR (Fig. 4g, h, i). In line with the expression levels of Nrf2 in the nucleus, the mRNA levels of *Nqo1*, *HO1*, and *Gclc* were significantly decreased in the TBI model group (*P* < 0.01), whereas the expression of these genes was significantly increased in the calcitriol treatment group (*P* < 0.01).

### The effects of calcitriol on TBI-induced oxidative damage

**Fig. 5:**
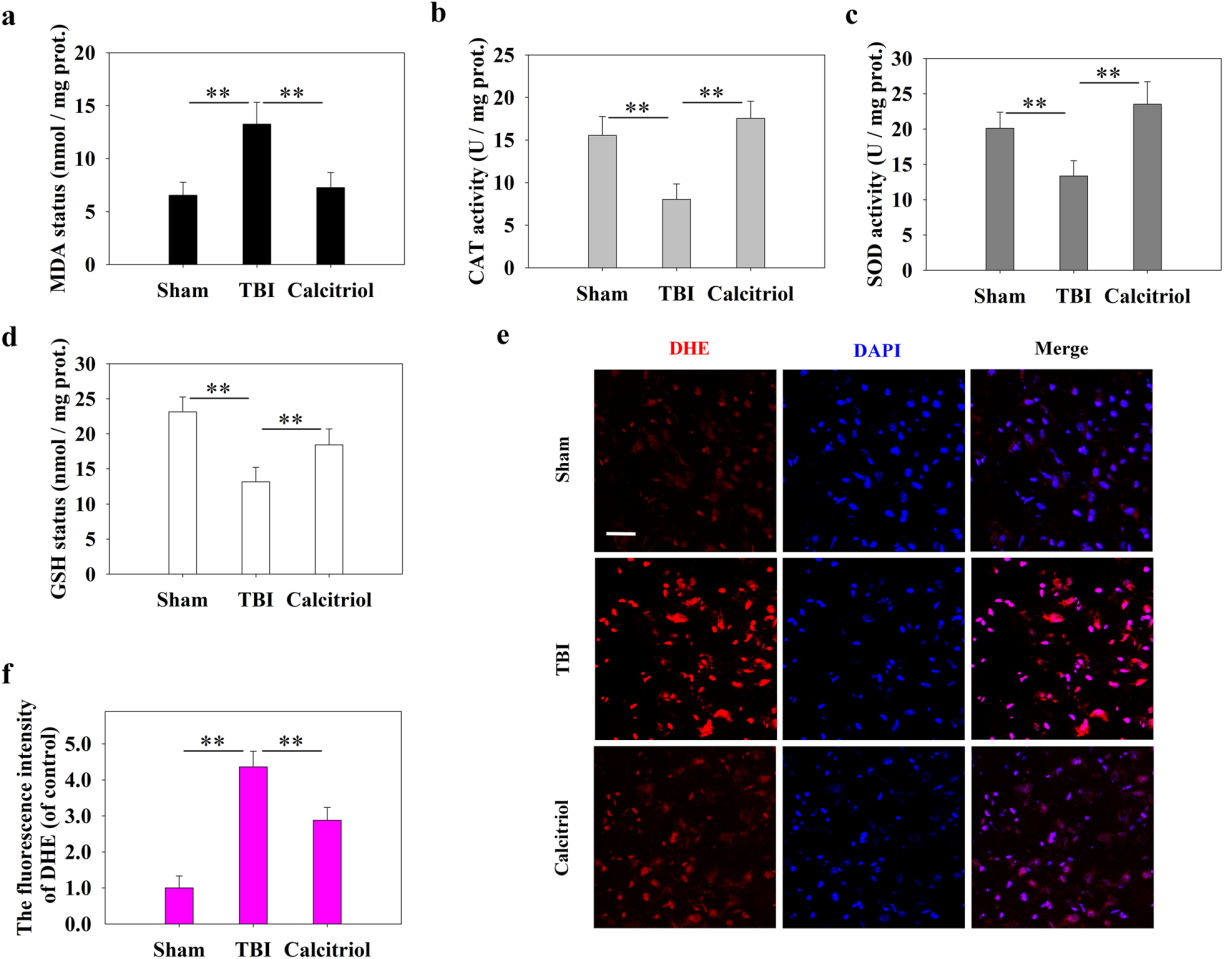
Calcitriol (0.5 µg/kg) treatment ameliorated TBI-induced oxidative stress. **a** Malondialdehyde (MDA) status. **b** Catalase (CAT) activity. **c** Superoxide dismutase (SOD) activity. **d** Glutathione (GSH) status. **e** Representative images of immunofluorescence assays of dihydroethidium (DHE) (Bar = 50 µm). **f** Statistical graphs of DHE-positive cells in different groups. Data are presented as means ± SEM (*n* = 5). **P < 0.01 versus the indicated groups. (Sham: sham-operated group; TBI: TBI model group; Calcitriol: TBI + calcitriol treatment group)

As shown in Fig. 5, while TBI caused significantly increased oxidative stress as evidenced by the increased lipid peroxidation product (MDA) levels (Fig. 5a) and decreased activity of antioxidant enzymes (SOD and CAT) and GSH status (Fig. 5b–d), calcitriol markedly ameliorated the altered oxidative disturbance, which is in line with the activated antioxidant Nrf2 signaling following calcitriol treatment. Additionally, calcitriol also reduced the TBI-induced ROS production as evidenced by the DHE immunofluorescence (Fig. 5e, f).

### The effects of inhibiting autophagy and knocking out Nrf2 on the neuroprotective activity of calcitriol

**Fig. 6:**
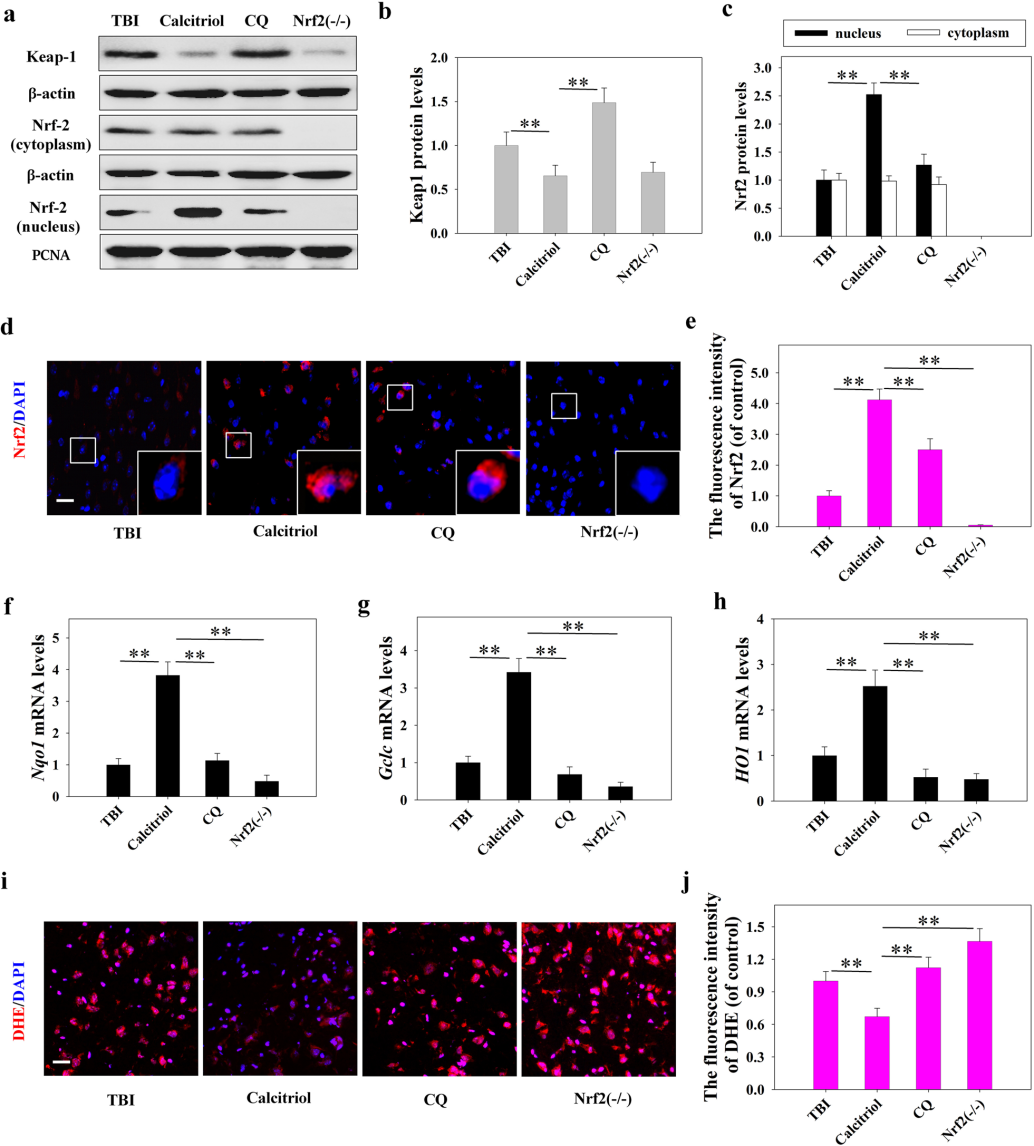
Inhibiting autophagy abolished calcitriol (0.5 µg/kg)-induced Nrf2 activation following TBI in mice. **a** Representative images of Western blot staining for Keap1, cytoplasmic Nrf2, and nuclear Nrf2. **b** Statistical graphs of Keap1 protein expression. **c** Statistical graphs of expression of Nrf2 in the nucleus and cytoplasm. **d**, **e** Representative immunofluorescence images and statistical graphs of Nrf2 translocation (Bar = 50 µm). **f** The relative mRNA expression level of *Nqo1*. **g** The relative mRNA expression level of *Gclc*. **h** The relative mRNA expression level of *HO1*. **i**, **j** Representative images and statistical graphs of immunofluorescence assays of dihydroethidium (DHE) (Bar = 50 µm). Data are presented as means ± SEM (*n* = 5). *P < 0.05 and **P < 0.01 versus the indicated groups. (TBI: TBI model group; Calcitriol: TBI + calcitriol treatment group; CQ: TBI + calcitriol + CQ treatment group; Nrf2^−/−^: TBI + calcitriol + *Nrf2* knockout group)

**Fig. 7:**
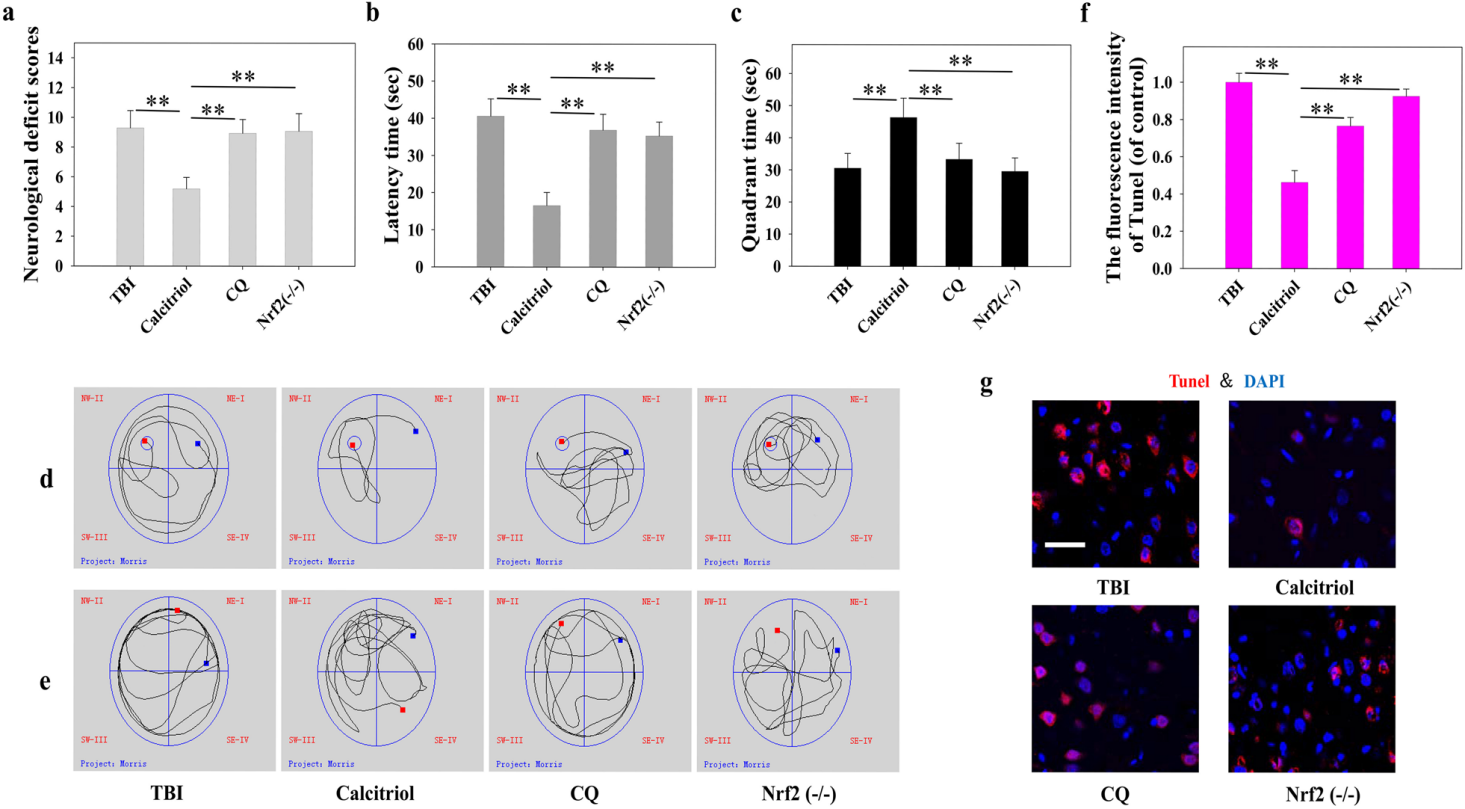
Blocking Nrf2 and autophagy abrogated the protective effects of calcitriol (0.5 µg/kg) on TBI-induced neurological dysfunction. **a** The variation in neurological deficits at 14 days after treatment was determined by neurological severity score tests. **b** The time (seconds) spent finding the submerged platform at 14 days. **c** The time (seconds) spent exploring the quadrant that initially contained the platform at 14 days. **d** Representative traces from the probe trials indicating the sample paths of the mice. **e** Representative traces after the platform was removed indicating the sample paths of the mice. **f** Statistical graphs of apoptotic cells (% of DAPI). **g** Representative immunofluorescence assays of TUNEL-positive cells (Bar = 50 µm). Data are presented as means ± SEM (*n* = 10). *P < 0.05 and **P < 0.01 versus the indicated groups. (TBI: TBI model group; Calcitriol: TBI + calcitriol treatment group; CQ: TBI + calcitriol + CQ treatment group; Nrf2^−/−^: TBI + calcitriol + *Nrf2* knockout group)

Our data demonstrated that both autophagy flux and the Keap1–Nrf2 pathway were activated by calcitriol treatment. Consequently, we assessed whether a noncanonical signaling network that includes both autophagy flux and the Keap1–Nrf2 pathway is induced by calcitriol. First, mice in the TBI + calcitriol treatment group were treated with CQ, a selective autophagy inhibitor that prevents autophagosome–lysosome fusion. As shown in Fig. 6, CQ treatment markedly suppressed the Keap1–Nrf2 pathway, inducing Keap1 protein expression and decreasing that of Nrf2 in the nucleus when compared with the TBI + calcitriol treatment group (*P* < 0.01). The levels of Nrf2 target genes were also examined. The mRNA levels of *Nqo1* and *Gclc* were significantly decreased with CQ treatment, whereas ROS production was significantly increased (*P* < 0.01 versus the calcitriol-only group). Interestingly, CQ treatment blocked the ameliorative effect of calcitriol on neurological behavior and suppressed neuronal apoptosis (Fig. 7), highlighting the critical role of autophagy flux in the neuroprotective effects of calcitriol. Additionally, *Nrf2*^*−/−*^ mice exhibited lower *Nqo1* and *Gclc* mRNA levels after calcitriol treatment (*P* < 0.01 versus the calcitriol-only treatment groups). As expected, the neuroprotection effect of calcitriol on TBI was abrogated in *Nrf2* knockout mice, which displayed markedly impaired neurological functions and neuronal apoptosis (Fig. 7). The neurological score evaluation and MWM hidden platform task data are depicted in Additional files 6 and 7.

### The effects of calcitriol on neuronal autophagy and Keap1-Nrf2 pathway in vitro

**Fig. 8:**
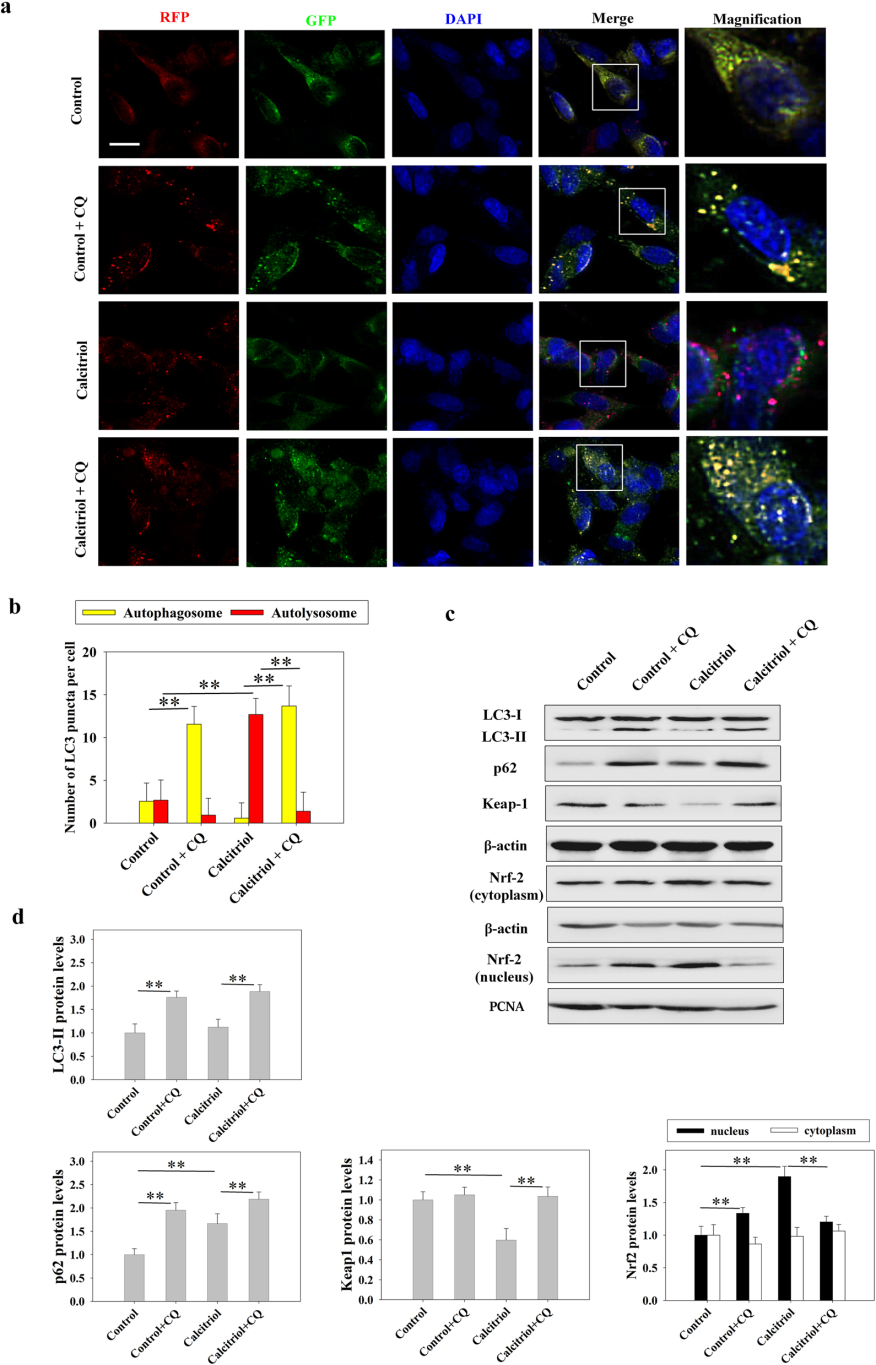
Effect of calcitriol (10 nM) on autophagy and Keap1-Nrf2 pathway in neuronal cells. **a** Representative immunofluorescence images of autophagy flux detected by a tandem RFP-GFP-LC3 reporter (Bar = 20 µm). **b** Statistical graphs of number of autophagosomes and autolysosomes per cell. **c** Representative images of Western blot staining for LC3, p62, Keap1, cytoplasmic Nrf2, and nuclear Nrf2. **d** Statistical graphs of LC3-II, p62, Keap1 and Nrf2 (cytoplasmic and nuclear) protein expression levels. Data are presented as means ± SEM (*n* = 5). *P < 0.05 and **P < 0.01 versus the indicated groups

We then used primary neurons from frontal cortex of fetal mouse to further confirm the molecular mechanism in vitro. The results from the MTT assay demonstrated that while calcitriol at concentration of 1 nM and 10 nM had no toxic effect, high concentration of calcitriol exposure (100 nM and 500 nM) significantly reduced cell viability when compared to the control group (*P* < 0.01). Therefore, the impact of calcitriol on Nrf2 signaling was assessed. It was observed that calcitriol (1–1000 nM) significantly increased nucleus Nrf2 expression compared to the control group (*P* < 0.01 for the 10–500 nM groups; *P* < 0.05 for the 1 nM group) (Additional file 8). Combining the results of the above experiments, we chose calcitriol with 10 nM concentration for further experiments to demonstrate the mechanism between autophagy and Nrf2 pathway in vitro. In order to show the autophagy flux, a tandem RFP-GFP-LC3 reporter was used. This assay takes advantage of differential pH sensitivity of GFP (acid labile) and RFP (acid resistant) fluorophores to assess acidification of autophagosomes (yellow) upon fusion with lysosomes (red only). As shown in Fig. 8, in cells treated with calcitriol, we observed accumulation of red autolysosomes compared to controls (*P* < 0.01). However, inhibition of autophagy flux by CQ caused impairment in autophagosome-lysosome fusion, displayed an increased accumulation of yellow autophagosomes (*P* < 0.01). These data confirmed that calcitriol treatment induced the activation of neuronal autophagic flux. Additionally, the expression levels of autophagy-related proteins were measured by Western blot. The level of p62 was higher in the calcitriol group than in the control group (*P* < 0.01). However, inhibition of autophagy flux by CQ induced the expression of LC3-II and p62 (*P* < 0.01). The expression of Keap1 was attenuated and the nuclear expression of Nrf2 was higher in the calcitriol treatment group than in control group (*P* < 0.01). However, inhibition of autophagy flux by CQ after calcitriol treatment markedly suppressed the Keap1–Nrf2 pathway, inducing Keap1 protein expression and decreasing Nrf2 status in the nucleus (*P* < 0.01).

## Discussion

Oxidative stress is the major cause of TBI-induced secondary injury, whereas Nrf2 is an important oxidative stress regulator in the protection of various cell types and organ systems. Recent studies have also shown that impairment of Nrf2 signaling in the injured brain may aggravate ROS production and cause oxidative stress, thereby inducing inflammatory response and cell death following TBI (Zhou et al. 2018; Dong et al. 2018; Bhowmick et al. 2019). However, the pathological mechanisms remain elusive and protective strategies for the oxidative brain damage are still limited.

VitD now is recognized as a pleiotropic secosteroid affecting multiple aspects of human physiology. The beneficial effects of VitD on CNS have been observed in animal models as well as in patients with cerebral ischemia, Alzheimer's disease, Parkinson's disease, and multiple sclerosis (Cui et al. 2017c; Di Somma et al. 2017). Our previous study has shown that activation of VDR is protective against neurological deficits and attenuates Nox2-induced oxidative stress in a rat model of TBI (Cui et al. 2017a). In line with these findings, the present research also found that treated with calcitriol, at doses ranging from 0.5 to 3 µg/kg, was effective in potentiating the recovery process in mice following TBI by mitigating neurological deficits, improving behavioral performance in the MWM test and retarding neuronal apoptosis. These findings support the beneficial effects of calcitriol in TBI and further demonstrate that the sustained treatment of calcitriol immediately after TBI may enhance the recovery process and prevent potential secondary injury. However, it should be noted that 3 µg/kg of calcitriol seems less protective than the lower doses, as evidenced by the probe test in MWM and neural apoptosis staining. The effect is probably because although calcitriol is neuroprotective against TBI, the drug itself may induce hypercalcemia and toxic effect. In line with our findings, previous research also showed that chronic treatment of 3 µg/kg of calcitriol induces weight loss and vascular calcification in rodents (Cass et al. 2012; Mori et al. 2020; Zelt et al. 2015). Additionally, we further demonstrated that while calcitriol can dose-dependently trigger Nrf2 activation, 100 nM and 500 nM calcitriol also reduced cell viability of neuronal cells, suggesting that high dose of calcitriol, while effective, may have been high enough to produce adverse side effects.

Nrf2 and autophagy are two critical stress responsive signaling pathways that are associated with redox balance. Nrf2 is a basic leucine zipper (CNC bZip) redox sensitive transcription factor that activates antioxidant response elements (AREs). The genes transcriptionally regulated by the AREs encode detoxification enzymes and antioxidant proteins thereby playing a central role in the oxidative stress modulation (Bhowmick et al. 2019). Notably, oxidative stress and autophagy are also intricately connected. In the past few years, autophagy has been proposed as a potential survival mechanism in the context of exaggerated ROS generation by timely removal of damaged and redundant substance, serving as a cytoprotective mechanism to restrain oxidative injury (Filomeni et al. 2015). Moreover, p62-dependent clearance of Keap1 has been found to regulate Nrf2 signaling and constitutes an important defense system against oxidative stress. p62 can bind and sequester Keap1, allowing the release of Nrf2 from Keap1 and Nrf2 translocation to the nucleus, thereby resulting in the activation of antioxidant genes (Dodson et al. 2015). As previously reported (Cui et al. 2017b), we found impaired autophagic flux in the injured brain with increased autophagosome accumulation. By using the lysosomal inhibitor, CQ, we confirmed the inhibited autophagic process following TBI as evidenced by the significant increase of LC3-II expression in Sham group but unchanged in TBI group following CQ treatment. Although autophagy was reported to be enhanced soon after acute brain injury to cope with the stress, it seems that prolonged time after TBI is likely to induce exhaustion of autophagy (Sarkar et al. 2014). Concomitantly, we observed a marked increase of Keap1 expression and a significant decrease of Nrf2 translocation, resulting in attenuated downstream antioxidant signaling and exaggerated oxidative stress following TBI. In parallel, calcitriol accelerated the recovery process and activated autophagic flux following TBI. The increased transcriptional level of ATG genes but decreased protein expression of LC3-II and p62 compared with TBI group indicates that calcitriol not only induced autophagosome formation but also facilitated its degradation. Meanwhile, calcitriol also triggered Nrf2 signaling, inducing the expression of targeted genes and protecting the brain from excessive oxidative stress. In support of our findings, recent studies also illustrate a pivotal role of Nrf2 in the protective effects of calcitriol or other analogs of the active form of VitD in Alzheimer's disease, lung injury, skin aging, liver failure and kidney toxicity (Zhang et al. 2019b; Saad El-Din et al. 2020; Abo El-Magd and Eraky 2020; Chen et al. 2019; Tao et al. 2019). Given that Keap-1 is targeted to autophagosomes for degradation, the reduction of Keap-1 expression and the decreased co-expression of p62 and Keap1 following calcitriol treatment suggest that Nrf2 might be activated by calcitriol through autophagy mediated by the interaction between p62 and Keap1. By using CQ to block autophagy, we found that cotreatment with CQ abrogated calcitriol-induced Keap-1 degradation and compromised Nrf2 signaling, resulting in the decreased downstream antioxidant genes and increased ROS production. As expected, both CQ treatment and Nrf2 genetic knockout abolished the beneficial effects of calcitriol on neurological function as well as its anti-apoptotic activity following TBI, indicating that the neuroprotective effect of calcitriol occurs through the interaction between autophagy and Nrf2 signaling. Additionally, we also observed a dose-dependent increase of Nrf2 signaling in neuronal cells following calcitriol exposure. Using the tandem fluorescent mRFP-GFP-LC3 adenovirus, we further proved that calcitriol can effectively trigger autophagic flux. Meanwhile, inhibiting autophagic process with CQ also blocked the calcitriol-induced Keap1 degradation and Nrf2 activation in vitro. These data collaboratively support the facilitating effect of calcitriol on autophagy and the activation of Nrf2 signaling. However, it should be noted that some researchers suggest that blocking lysosomal function would lead to autophagosome accumulation and thereby promote p62 to bind with Keap1, sequestering it from Nrf2 and resulting in Nrf2 translocation (Park et al. 2019; Fan et al. 2018). On the other hand, there is also accumulating evidence which is in accordance with our findings, demonstrating that autophagy-induced Keap1 degradation can be effectively abrogated by the lysosomal inhibitors, CQ and Bafilomycin A1, which would accelerate oxidative stress or compromised the protective effects of various interventions in the brain, heart and liver tissues (Deng et al. 2020; Tan et al. 2020; Lee et al. 2020). These discrepancies might be attributed to the different cell types or disease models that may have various basal autophagic condition, and because of the different doses and treatment duration that may cause diverse autophagic inhibitory status. Following TBI or other stimuli, the injury progression and ROS production last even for weeks. To this end, the calcitriol-induced continuously autophagic clearance of Keap-1 and constantly active Nrf2 signaling are critical to mitigate the oxidative damage. In this study, CQ was co-treated with calcitriol, which may induce persistent inhibition on this process and thereby exacerbate brain damage.

## Conclusions

**Fig. 9:**
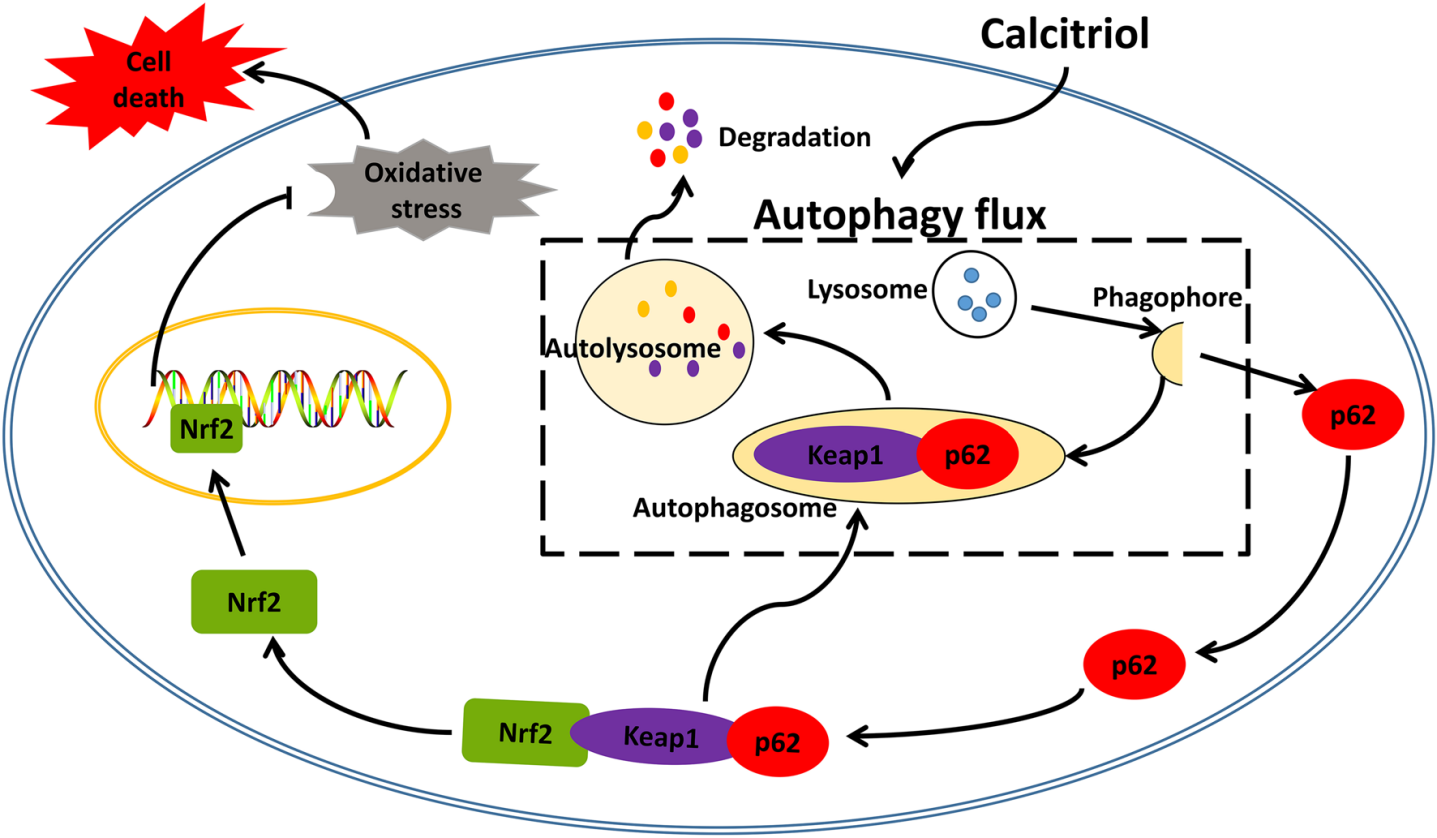
The molecular mechanisms underlying the neuroprotective effects of calcitriol against TBI. Calcitriol maintains redox balance and protects the brain from oxidative damage through Nrf2 signaling mediated by the autophagic degradation of Keap1

Taken together, the present study firstly demonstrated that autophagic flux serves as an upstream regulator required for calcitriol-induced Nrf2 activation, thereby alleviating oxidative damage and cell apoptosis following TBI (Fig. 9). Additionally, our data demonstrated that autophagy and Nrf2, the two critical pathways in maintaining homeostasis and redox balance, are intricately connected and both are indispensable for the antioxidant and anti-apoptosis actions of calcitriol. These findings would further enrich and shed novel insight on the neurological functions of calcitriol, providing an interesting target for the autophagic dysfunction and oxidative stress following TBI-induced neuronal damage.

## Supplementary Information

**Additional file 1: Additional Table 1.** Neurological Severity Scores (NSS).

**Additional file 2: Additional Table 2.** Primer sequences used for the qPCR analysis.

**Additional file 3.** The immunofluorescence images of the negative control (secondary antibodies only) (Bar = 50 µm).

**Additional file 4.** Neuroprotective effects of calcitriol in TBI-induced neurological deficits. The variation of neurological deficits at 1–14 days was determined by neurological severity score tests. Data are presented as means ± SEM (*n* = 10). *P < 0.05 and **P < 0.01 versus the indicated groups.

**Additional file 5.** Neuroprotective effects of calcitriol in TBI-induced memory dysfunction. **a** The variation of Time (seconds) spent in finding the submerged platform at 11–14 days was determined by MWM tests. **b** There were no significant differences in swim speeds among groups. Data are presented as means ± SEM (*n* = 10). *P < 0.05 and **P < 0.01 versus the indicated groups.

**Additional file 6.** Blocking Nrf2 and autophagy abrogated the beneficial effects of calcitriol (0.5 µg/kg) on TBI-induced neurological deficits. The variation of neurological deficits at 1–14 days was determined by neurological severity score tests. Data are presented as means ± SEM (*n* = 10). *P < 0.05 and **P < 0.01 versus the indicated groups.

**Additional file 7.**Autophagic inhibition and Nrf2 genetic knockout abrogated the beneficial effects of calcitriol (0.5 µg/kg) on TBI-induced memory dysfunction. **a** The variation of Time (seconds) spent in finding the submerged platform at 11–14 days was determined by MWM tests. **b** There were no significant differences in swim speeds among groups. Data are presented as means ± SEM (*n* = 10). *P < 0.05 and **P < 0.01 versus the indicated groups.

**Additional file 8.** Effect of calcitriol on cell viability and Nrf2 protein levels in vitro. **a** Statistical graphs of the variation in cell viability detected by MTT assay. **b** **c** Representative images and statistical graphs of Western blot staining for cytoplasmic Nrf2 and nuclear Nrf2 after different dosages of calcitriol treatment. Data are presented as means ± SEM (*n* = 5). *P < 0.05 and **P < 0.01 versus the indicated groups.

## Data Availability

Data are available upon reasonable request. The data used in the current study are available from the corresponding author on reasonable request.

## Abbreviations

ATG genes: Autophagy-related genes
CAT: Catalase
CCI: Controlled cortical impact
CQ: Chloroquine
DHE: Dihydroethidium
GSH: Glutathione
H&E: Hematoxylin and eosin
Keap1: Kelch-like ECH-associated protein 1
MDA: Malondialdehyde
MWM: Morris water maze
Nrf2: Nuclear factor E2-related factor 2
PBS: Phosphate-buffered saline
ROS: Reactive oxygen species
SOD: Superoxide dismutase
TBI: Traumatic brain injury
TUNEL: Terminal deoxynucleotidyl transferase-mediated cyanine–dUTP nick-end labeling
VDR: Vitamin D receptor
VitD: Vitamin D
