# Supplementary material for: Calcitriol confers neuroprotective effects in traumatic brain injury by activating Nrf2 signaling through an autophagy-mediated mechanism
Source: Mol Med. 2021 Sep 23;27:118. doi: 10.1186/s10020-021-00377-1 (PMC8461874; doi:10.1186/s10020-021-00377-1)
Supplement: Supplementary file 1 — Additional file 1: Additional Table 1. Neurological Severity Scores (NSS). [file 10020_2021_377_MOESM1_ESM.docx]

**Additional Table1.** Neurological Severity Scores (NSS)

|  | **Score** |
| --- | --- |
| **Raising mouse by tail(normal=0; maximum=3)** | **(3)** |
| Flexion of forelimb | 1 |
| Flexion of hindlimb | 1 |
| Head moved >10° to vertical axis within 30s | 1 |
| **Placing mouse on floor** **(normal=0; maximum=3)** | **(3)** |
| Normal walk | 0 |
| Inability to walk straight | 1 |
| Circling toward paretic side | 2 |
| Falls down to paretic side | 3 |
| **Sensory tests(normal=0; maximum=2)** | **(2)** |
| Placing test (visual and tactile test) | 1 |
| Proprioceptive test (deep sensation) | 1 |
| **Beam balance tests** **(normal=0; maximum=6)** | **(6)** |
| Balances with steady posture | 0 |
| Grasps side of beam | 1 |
| Hugs beam and 1 limb falls down from beam | 2 |
| Hugs beam and 2 limbs fall down from beam, or spins on beam (>60s) | 3 |
| Attempts to balance on beam but falls off (>40s) | 4 |
| Attempts to balance on beam but falls off (>20s) | 5 |
| Falls off; no attempt to balance or hang on to beam (<20s) | 6 |
| **Reflex absence and abnormal movements** | **(4)** |
| Pinna reflex (head shake when auditory meatus is touched) | 1 |
| Corneal reflex (eye blink when cornea is lightly touched with cotton) | 1 |
| Startle reflex (motor response to a brief noise) | 1 |
| Seizures, myoclonus, myodystony | 1 |
| **Maximum points** | **(18)** |
