# Supplementary material for: Calcitriol confers neuroprotective effects in traumatic brain injury by activating Nrf2 signaling through an autophagy-mediated mechanism
Source: Mol Med. 2021 Sep 23;27:118. doi: 10.1186/s10020-021-00377-1 (PMC8461874; doi:10.1186/s10020-021-00377-1)
Supplement: Supplementary file 2 — Additional file 2: Additional Table 2. Primer sequences used for the qPCR analysis. [file 10020_2021_377_MOESM2_ESM.docx]

**Additional Table 2** Primer sequences used for the qPCR analysis

| Gene | Sense Primer (5’-3’) | Antisense Primer (5’-3’) length (bp) |
| --- | --- | --- |
| LC3  P62  Beclin 1  NQO-1  GCLC  HO-1 | CGTCACCCAGGCGAGTTACC  GGAGGAGACGATGACTGG  AGATTGGACCAGGAGGAA  TACGATCCTCCCTCAACA  TGGCAGACAATGAGGTTT  GGCAGAGGGTGATAGAAGAGG | GAGATGCGTCTGCGGTTCG 114  TCTGTAGGAGCCTGGTGAG 145  AGGTGGCATTGAAGACAT 160  TACAGCAGCCTCCTTCAT 108  ACAGCGGAATGAGGAAGT 175  AGCTCCTGCAACTCCTCAAA 182 |
| β-actin | CATCCTGCGTCTGGACCTGG | TAATGTCACGCACGATTTCC 116 |
