# Supplementary figures and images for: Calcitriol confers neuroprotective effects in traumatic brain injury by activating Nrf2 signaling through an autophagy-mediated mechanism
Source: Mol Med. 2021 Sep 23;27:118. doi: 10.1186/s10020-021-00377-1 (PMC8461874; doi:10.1186/s10020-021-00377-1)

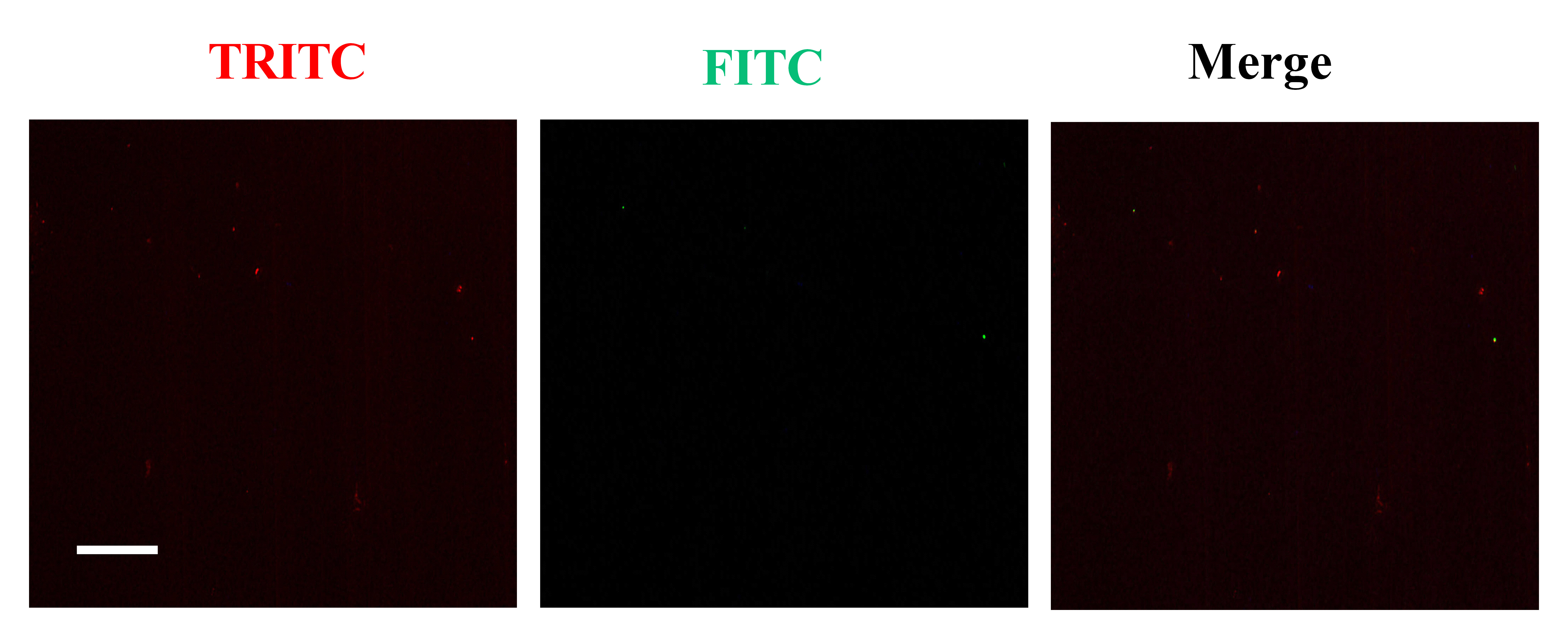

Supplement: Supplementary file 3 — Additional file 3. The immunofluorescence images of the negative control (secondary antibodies only) (Bar = 50 µm). [file 10020_2021_377_MOESM3_ESM.jpg]

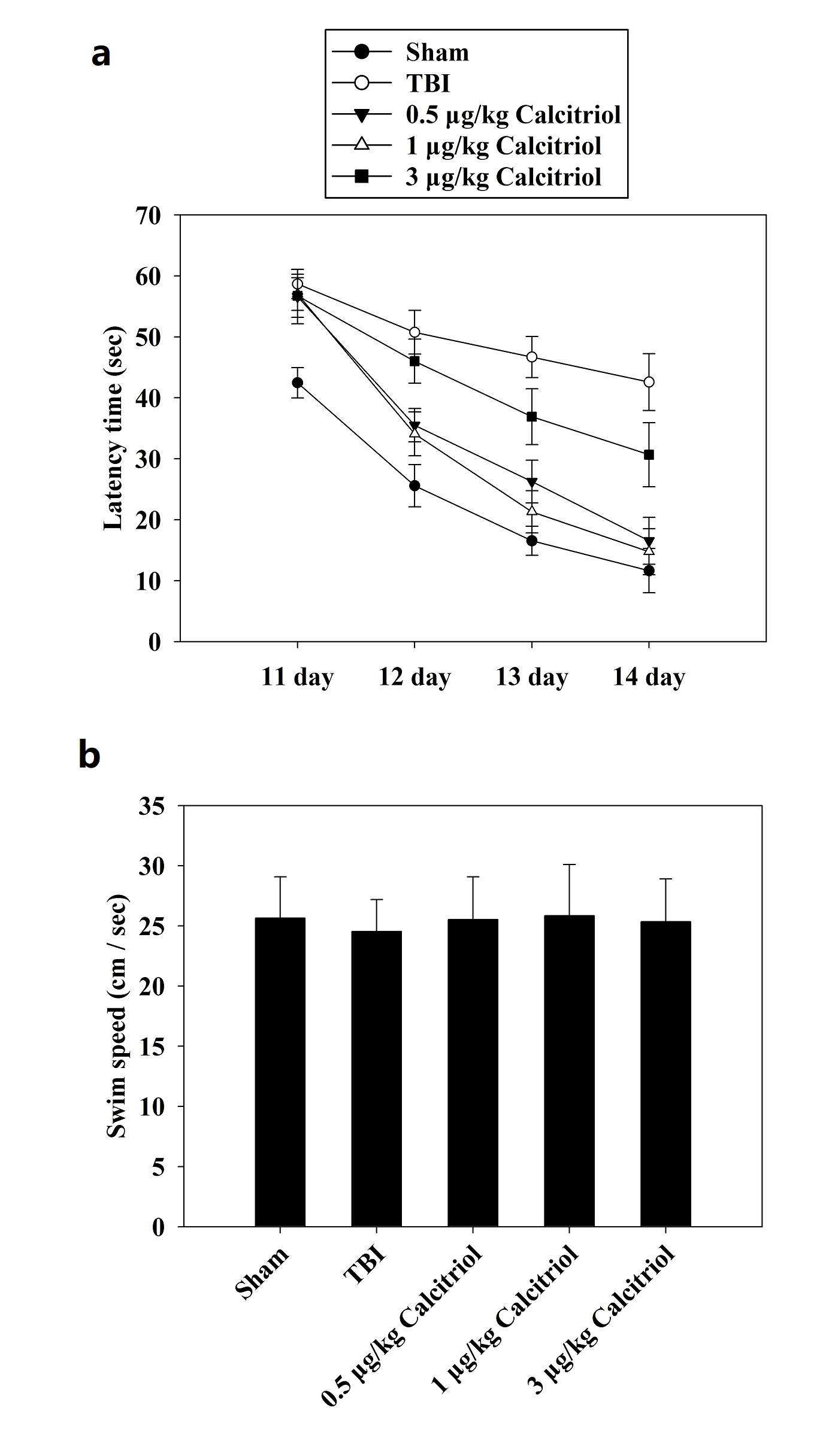

Supplement: Supplementary file 5 — Additional file 5. Neuroprotective effects of calcitriol in TBI-induced memory dysfunction. a The variation of Time (seconds) spent in finding the submerged platform at 11–14 days was determined by MWM tests. b There were no significant differences in swim speeds among groups. Data are presented as means ± SEM (n = 10). *P < 0.05 and **P < 0.01 versus the indicated groups. [file 10020_2021_377_MOESM5_ESM.tif]

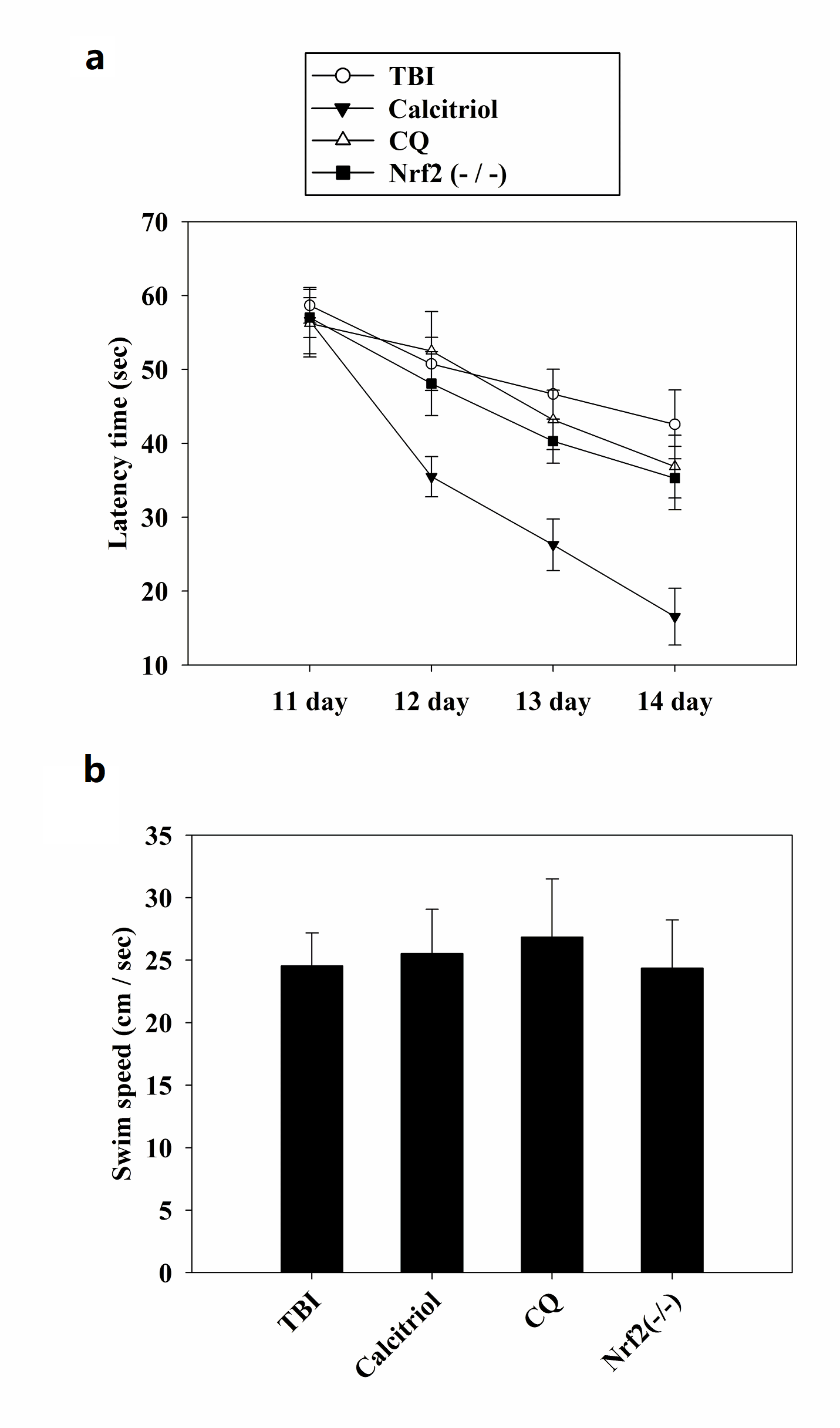

Supplement: Supplementary file 7 — Additional file 7.Autophagic inhibition and Nrf2 genetic knockout abrogated the beneficial effects of calcitriol (0.5 µg/kg) on TBI-induced memory dysfunction. a The variation of Time (seconds) spent in finding the submerged platform at 11–14 days was determined by MWM tests. b There were no significant differences in swim speeds among groups. Data are presented as means ± SEM (n = 10). *P < 0.05 and **P < 0.01 versus the indicated groups. [file 10020_2021_377_MOESM7_ESM.tif]
